# P-543. A Descriptive Report of Discontinuation Rates, Blips, and Viremia Associated with Cabotegravir/Rilpivirine at One Ryan White HIV/AIDS Program-funded site in Northern Virginia

**DOI:** 10.1093/ofid/ofae631.742

**Published:** 2025-01-29

**Authors:** Caitlin Prather, Katie Richey, John Paul Verderese

**Affiliations:** Inova Health System, Fairfax, Virginia; Inova Health System, Fairfax, Virginia; Inova Health System, Fairfax, Virginia

## Abstract

**Background:**

Cabotegravir/rilpivirine (CAB/RPV) is the first and only long-acting complete regimen for the treatment of HIV-1 infection. Rates of discontinuation, viral blips, and viremia were low in Phase III clinical trials for CAB/RPV, but data from additional locations in real-world conditions remains lacking. This descriptive report explores the frequency of these occurrences in patients treated with CAB/RPV at one Ryan White HIV/AIDS Program (RWHAP)-funded site.

**Methods:**

This was a single-center, retrospective, observational chart review of people living with HIV at one institution in Northern Virginia. Patients were included if they received at least one dose of injectable CAB/RPV between January 2021 and April 2024. Data collected from the electronic medical record included demographics, body mass index (BMI), history of antiretroviral drug mutations, HIV viral load, and HIV treatment history.

**Results:**

A total of 107 patients were included in this study with a mean age of 45 years. Of these, 2% were assigned female at birth and 49% were Black. In the study period, 16% of patients discontinued the medication. The most common causes for discontinuation were adverse effects, insurance denials, patient preference for oral therapy, and frequently missing appointments. Of the 17 patients who discontinued therapy, only one was discontinued due to virologic failure. As of April 2024, 85 patients remain on CAB/RPV. Of these, 43% have experienced at least one blip, 25% have experienced low-level viremia, and 61% have experienced either a blip or low-level viremia since starting CAB/RPV. Duration of therapy ranged from 1 to 1020 days (average 579 days) since first injection and longer duration of therapy weakly correlated with prevalence of blips or viremia. 92% of doses have been administered within the allowable 14-day treatment window. There was no significant difference between BMI at the start of treatment to April 2024.

**Conclusion:**

Among patients treated with CAB/RPV, viral blips and low-level viremia are common but are not associated with treatment discontinuation or treatment failure. Rates of discontinuation due to adverse effects were higher than what was observed in Phase III randomized controlled trials of CAB/RPV, though treatment failure was uncommon as expected.

**Disclosures:**

**All Authors**: No reported disclosures

